# Effect of Intraoperative Local Administration of Tranexamic Acid on Hemorrhage in Patients Undergoing Open Prostatectomy: A Double-Blinded Randomized Parallel-Group Trial

**DOI:** 10.1155/aiu/9294177

**Published:** 2025-08-04

**Authors:** Mahdi Hemmati Ghavshough, Zahra Shirinzadeh, Mansour Alizadeh, Mohammad Sadri, Saman Farshid

**Affiliations:** ^1^Urology Department, Rutgers Cancer Institute, New Brunswick, New Jersey, USA; ^2^Epidemiology Department, School of Public Health of Tehran University, Tehran, Iran; ^3^Clinical Research Development Unit of Imam Khomeini Hospital, Urmia University of Medical Sciences, Urmia, Iran

**Keywords:** hemorrhage, open prostatectomy, tranexamic acid

## Abstract

**Background and Objective:** Benign prostatic hyperplasia (BPH) often necessitates surgical treatment, with open prostatectomy remaining a standard approach. However, this procedure carries a significant risk of intraoperative and postoperative bleeding, often requiring blood transfusions. Tranexamic acid (TXA), an antifibrinolytic agent, has shown potential in reducing surgical blood loss. This study aims to evaluate the effect of intraoperative local administration of TXA on perioperative blood loss in patients undergoing open prostatectomy for BPH.

**Methods:** In this double-blind randomized controlled trial, 140 patients with BPH were assigned to either a TXA group or control group. In the intervention group, 1 g of TXA was diluted in 100 mL of normal saline and injected into the prostatic fossa during surgery, followed by three additional postoperative doses. The primary outcome was total perioperative blood loss. Secondary outcomes included changes in hemoglobin, hematocrit, platelet count, transfusion requirement, and length of hospital stay. Baseline differences, including a significant age gap between the groups (mean age: TXA group 60.70 ± 7.44 years vs. control group 70.50 ± 6.68 years), were statistically adjusted during analysis.

**Results:** Perioperative blood loss was significantly lower in the TXA group (116.65 ± 43.23 mL) compared to the control group (210.27 ± 87.94 mL, *p* value = 0.001). The mean hemoglobin drop was also significantly reduced in the TXA group at both 24 and 48 h postoperatively. Fewer patients in the TXA group required blood transfusion (2.85%) compared to the control group (10%, *p* value = 0.03). No major adverse events directly attributed to TXA were identified, although one patient in the TXA group developed a pulmonary embolism.

**Conclusion:** Intraoperative local administration of TXA significantly reduces perioperative blood loss and the need for blood transfusion in patients undergoing open prostatectomy. TXA appears to be a safe and effective strategy for minimizing surgical bleeding in this setting.

**Trial Registration:** Iranian Registry of Clinical Trials: IRCT20180625040232N8

## 1. Introduction

Benign prostatic hyperplasia (BPH) is a common condition in older men, with approximately 50% of men aged 60 and nearly 90% of those aged 85 exhibiting histopathological changes associated with this disorder [1]. In moderate to severe cases, surgical intervention is often required for effective management. Among surgical options, open simple prostatectomy remains a standard treatment for large-volume BPH when minimally invasive techniques are not suitable [2]. However, this procedure carries a significant risk of complications, particularly intraoperative and postoperative bleeding. Reported blood loss during open prostatectomy ranges from 700 to 1500 mL, with nearly 10% of patients who require blood transfusions due to perioperative hemorrhage [3].

Several factors contribute to postoperative bleeding in open prostatectomy, including resected tissue weight, preoperative urine culture, patient age, prostate size, operation duration, anesthesia type, preoperative finasteride use, and blood pressure [4]. Additionally, increased urinary fibrinolytic activity following surgery has been linked to elevated levels of plasminogen in the urothelium and urine, which can accelerate clot degradation [5].

To address these challenges, various strategies have been employed to minimize operative bleeding, including intravenous estrogen, intraprostatic vasopressin, early catheter removal, and preoperative finasteride administration [5–7]. Among these, tranexamic acid (TXA) has garnered increasing attention for its hemostatic potential [8]. A systematic review by Longo et al. demonstrated that TXA significantly reduces intraoperative blood loss and transfusion rates during open prostatectomy without elevating the risk of thromboembolic events [9]. TXA, a synthetic lysine derivative, acts as an antifibrinolytic by binding to plasminogen and inhibiting its interaction with fibrin, thereby stabilizing clot formation [10]. By preventing fibrinolysis, TXA may effectively reduce both intraoperative and postoperative bleeding.

This study aims to evaluate the efficacy of TXA in reducing perioperative and postoperative blood loss during open prostatectomy for BPH. Specifically, we assess its impact on intraoperative bleeding, transfusion requirements, and recovery parameters.

We hypothesize that intraoperative local administration of TXA significantly reduces total blood loss compared to standard care. Furthermore, we anticipate that TXA-treated patients will require fewer postoperative transfusions and experience improved recovery outcomes. Ultimately, this research seeks to provide robust evidence supporting the integration of TXA into standard routine surgical management of large-volume BPH.

## 2. Methods

### 2.1. Study Design and Population

This study was conducted at the Urology Department of Imam Khomeini Educational and Medical Hospital, affiliated with Urmia University of Medical Sciences, between September 2020 and December 2022. It was a prospective, randomized, double-blind, placebo-controlled pilot clinical trial involving 140 adult male patients diagnosed with BPH. Eligible patients were classified as ASA physical status I-II and identified as candidates for open prostatectomy based on clinical evaluation, biopsy, and MRI findings. The study protocol was approved by the ethics committee (IR.UMSU.REC.1400.030). Written informed consent was obtained from all participants in accordance with institutional and national guidelines.

### 2.2. Inclusion and Exclusion Criteria

The inclusion criteria were as follows:

• Male patients diagnosed with BPH at Imam Khomeini Hospital between 2013 and 2023.
• Scheduled for open simple prostatectomy.
• Age between 18 and 90 years.
• Body mass index (BMI) less than 35.
• Surgery performed by an urologist with experience of more than 50 prostatectomy cases.

The exclusion criteria were as follows:

• History of thrombotic events (e.g., myocardial infarction [MI], stroke, deep venous thrombosis [DVT], and pulmonary embolism [PE]) within 6 months prior to surgery.
• Patients scheduled for Robot-Assisted Radical Prostatectomy (RARP) without pelvic lymphadenectomy.
• Age over 90 years.
• Surgeon with fewer than 50 cases.
• Known sensitivity or contraindication to TXA.
• Coagulation disorders (e.g., anemia, hemophilia, leukemia, lymphoma, myeloma, and Leiden mutation).
• Postoperative hypotension.
• Severe cardiac disease based on preoperative cardiac risk assessment.
• Chronic kidney diseases (serum creatinine > 180 μmol/L).
• Use of anticoagulants (e.g., warfarin, aspirin, and dipyridamole).
• Elevated partial thromboplastin time (aPTT) and prothrombin time (PT).
• Participation in another clinical trial.

### 2.3. Sample Size Calculation

The sample size was calculated based on an anticipated mean difference of 200 (mL) in perioperative blood loss between groups, assuming a standard deviation of 400 (mL). With a significance level of 0.05 and a power of 80%, a minimum of 63 patients per group was required. To account for potential dropouts, the sample size was increased to 70 per group, totaling 140 participants.

### 2.4. Procedure (Intervention and Data Collection)

Of 148 patients initially screened, 8 were excluded, yielding 140 participants. Randomization was performed using a block randomization method via https://www.randomizer.org. Each patient was assigned a unique random number, and allocation was concealed in sealed, opaque envelopes. Patients were randomized in a 1:1 ratio to the TXA or control group (70 per group). The trial was double-blinded: Neither patients, surgeons, nor assessors were aware of group assignments. A designated nurse prepared the infusion set using the allocation envelope on the day of surgery. In the TXA group, a total of four 1 g doses of TXA were administered. The first dose consisted of 1 g of TXA diluted in 100 mL of normal saline and injected directly into the prostate fossa intraoperatively after adenoma enucleation. Following surgery, three additional 1 g doses were administered sequentially within the first 6 h postoperatively.

1. Via the Foley catheter into the bladder
2. Injected into the prostatic fossa through the surgical drain
3. Mixed with 250 mL of irrigation fluid and applied during postoperative bladder irrigation.

This local multisite administration protocol was designed to ensure prolonged exposure of TXA to the surgical field, bladder mucosa, and irrigation system, maximizing antifibrinolytic effects while minimizing systemic absorption. The control group received an identical placebo in appearance and volume to maintain blinding. A modified anastomosis technique using two V-Loc sutures was employed to facilitate early continence. A single dose of a broad-spectrum aminopenicillin was given as prophylaxis.

### 2.5. Variable and Outcomes

Baseline included age, weight, height, BMI, prostate volume, and prostate-specific antigen (PSA) levels. The primary outcome was perioperative blood loss, calculated using the following validated formula:

Actual blood loss (mL) = ((body weight (kg) × mean blood volume (mL/kg)) × initial Hb (g/dL) − final Hb (g/dL)])/(average initial and final Hb (g/dL)).

Blood volume was estimated at 70 mL/kg for adult males.

Secondary outcomes included:

• Hemoglobin (Hb), hematocrit (HCT), and platelet (PLT) counts at 24 and 48 h postoperatively.
• Blood transfusion rate.
• Duration of surgery.
• Length of hospital stay.
• Volume of irrigation fluid used.
• Type of anesthesia.
• Incidence of thromboembolic events (DVT or PE), assessed clinically and with D-dimer testing.

Intraoperative fluid replacement and transfusion were factored into blood loss estimates. To reduce intraoperative bleeding risk from inflammation or adhesions, a minimum interval of 6 weeks was observed between prostate biopsy and surgery.

### 2.6. Ethics and Reporting Standards

The study received approval from the institutional review board, and all participants gave written informed consent. Study conduct adhered to CONSORT guidelines for randomized controlled trials to ensure transparency and completeness in reporting.

### 2.7. Statistical Analysis

Data analysis was conducted using SPSS 21, with categorical and continuous data presented as mean ± standard deviation and frequency (percentage). A paired sample *t*-test was employed to compare mean values of continuous variables before and after the intervention, while analysis of covariance (ANCOVA) was utilized for between-group comparisons of mean values postintervention, adjusting for baseline values. The chi-squared test was used to compare categorical data between groups. Additionally, independent samples *t*-test, Mann–Whitney *U* test, or Kruskal–Wallis's test was applied to compare continuous variables between groups. For categorical variables with small expected frequencies, such as transfusion requirement, Fisher's exact test was used instead of the chi-square test to ensure valid inference. Linear regression was employed to predict the 24- and 48-h Hb drop, accounting for estimated bias. The sign of a linear regression coefficient indicated the positive or negative correlation between each independent variable and the dependent variable. A *p* value < 0.05 was considered statistically significant.

## 3. Results

### 3.1. Study Flow and Baseline Characteristics

**Figure 1:**
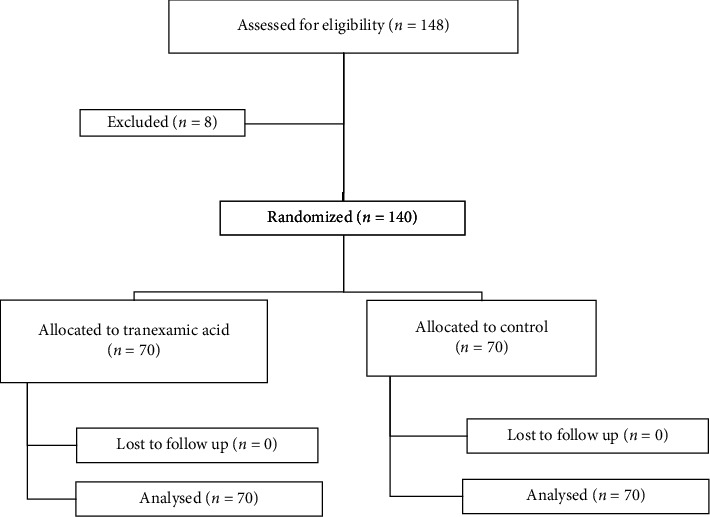
CONSORT flowchart diagram.

In this double-blind clinical trial, 148 patients with urinary symptoms due to BPH who were candidates for open prostatectomy were screened. Of these, 140 patients met the inclusion criteria and agreed to participate. They were randomly allocated into two equal groups of 70 patients each. No sample attrition occurred during the study (Figure 1).

### 3.2. General Perioperative Data

The mean age, weight, height, and BMI in the TXA group were 60.07 ± 7.44 years, 74.8 ± 2.2 kg, 173.2 ± 3.9 cm, and 25.04 ± 3.44 kg/cm^2^, respectively. In the control group, these corresponding values were 70.50 ± 6.68 years, 80.5 ± 2.7 kg, 176.3 ± 4.5 cm, and 26.04 ± 4.22 kg/cm^2^. No significant differences were observed between groups for weight (*p* value = 0.326), height (*p* value = 0.459), or BMI (*p* value = 0.125) (Table 1). Although the difference in age was not statistically significant (*p* value = 0.234), the younger average age in the TXA group may represent a potential confounder.

The mean duration of hospitalization was 2.50 days in the TXA group and 2.20 days in the control group (*p* value = 0.06) (Table 1). The mean prostate volume in the TXA group and control groups was 97.72 ± 40.86 cc and 96.24 ± 34.32 cc, respectively (*p* value = 0.817) (Table 1). The mean total PSA was 6.23 ± 4.58 ng/dL in the TXA group and 5.83 ± 2.15 ng/dL in the control group (*p* value = 0.064) (Table 1).

### 3.3. Surgery-Related Indicators

The mean resected tissue weight was 16.73 ng/dL in the TXA group and 16.54 ng/dL in the control group (*p* value = 0.452) (Table 1). The average volume of irrigating fluid absorbed was 49.57 ± 7.88 L in the TXA group and 50.48 ± 9.24 L in the control group (*p* value = 0.53) (Table 1). The mean surgery duration in the TXA group was 62.11 ± 5.42 min, and in the control group, it was 57.03 ± 8.12 min (*p* value = 0.344) (Table 1).

The estimated mean blood loss was significantly lower in the TXA group (116.65 ± 43.23 mL) compared to the control group (210.27 ± 87.94 mL) (*p* value = 0.001). Blood loss per gram of resected tissue was also significantly lower in the TXA group (6.97 mL/g vs. 12.69 mL/g, *p* value = 0.01). Blood loss was estimated using a validated Hb-balance formula based on changes in Hb concentration, body weight, and blood volume. This method provides a more physiologic estimate than visual or volumetric techniques.

Regarding anesthesia type, 1.4% of patients received general anesthesia, 0.7% received epidural anesthesia, and 97.9% received spinal anesthesia.

### 3.4. Blood-Related Indicators

**Figure 2:**
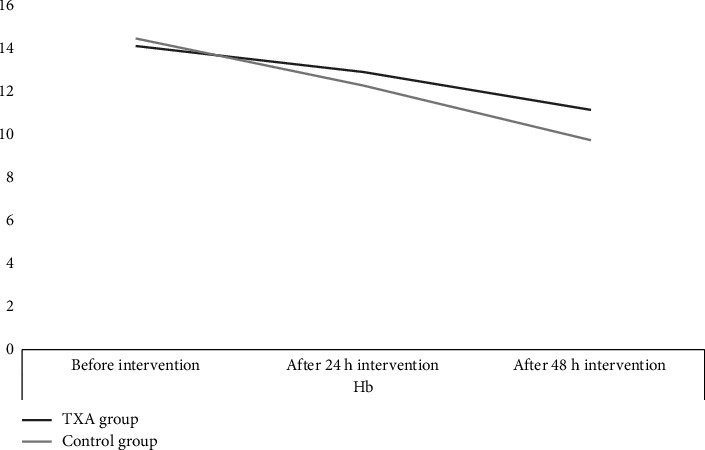
Hemoglobin changes in 24 and 48 h after intervention.

Preoperative mean Hb was 14.12 ± 1.34 g/dL in the TXA group and 14.47 ± 1.56 g/dL in the control group (*p* value = 0.316), indicating no significant baseline difference (Table 2). At 24 h postoperatively, Hb decreased to 12.91 ± 1.05 g/dL in the TXA group and 12.28 ± 1.23 g/dL in the control group (*p* value = 0.214). At 48 h, Hb levels were 11.15 ± 1.54 g/dL in the TXA group and 9.74 ± 1.02 g/dL in the control group, with a statistically significant difference (*p* value = 0.02) (Table 2). However, a more accurate comparison is reflected in the magnitude of Hb drop. The mean reduction in Hb was significantly greater in the control group than in the TXA group at both time points. At 24 h, the Hb drop was −1.19 ± 0.99 g/dL in the TXA group versus −2.194 ± 1.43 g/dL in the control group (*p* value = 0.0001), and at 48 h it was −1.78 ± 0.97 and −2.79 ± 1.04 g/dL, respectively (*p* value = 0.0001) (Table 3) (Figure 2).

**Figure 3:**
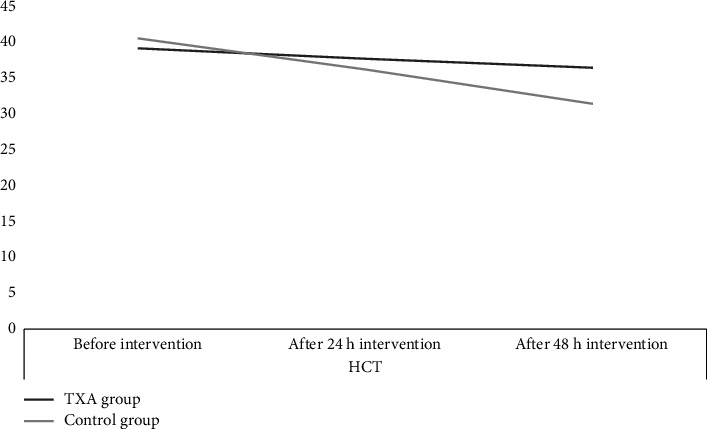
Hematocrit changes in 24 and 48 h after intervention.

Preoperative HCT in the TXA group and the control group was 39.13% ± 3.53% and 40.52% ± 2.89%, respectively (*p* value = 0.256). At 24 h after the intervention, HCT was 37.67% ± 5.65% and 36.19% ± 4.87%, respectively (*p* value = 0.162). At 48 h, a significant difference emerged: 36.42% ± 4.02% in the TXA group vs 31.40% ± 5.75% in the control group (*p* value = 0.01). In the TXA group and the control group, the mean HCT significantly decreased to 1.46 ± 0.86 and 4.33 ± 1.53% 24 h after the intervention (*p* value = 0.0003) and 1.25 ± 0.92 and 4.79 ± 1.42% 48 h after the intervention (*p* value = 0.0004) (Figure 3).

**Figure 4:**
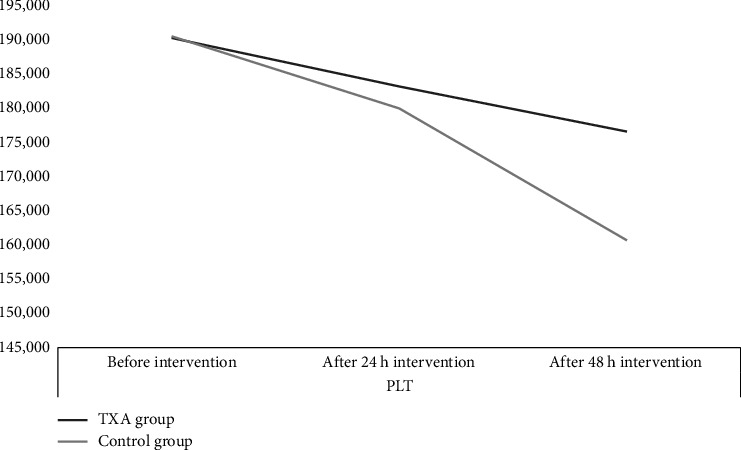
Platelet changes in 24 and 48 h after intervention.

Preoperative PLT counts were 190,275.5 ± 59,127 and 190,519.6 ± 57,319 plts/μL in the TXA and control groups, respectively (*p* value = 0.204). At 24 h, PLT counts were 183,195.1 ± 48,760 and 179,965.6 ± 50,087 plts/μL (*p* value = 0.197) and at 48 h, 176,588.6 ± 49,654 and 160,687.3 ± 59,761 plts/μL (*p* value = 0.01) for the TXA and control groups, respectively. The PLT counts in the TXA group and the control group significantly reduced to 7080.4 ± 326 and 10,554.0 ± 489 plts/μL 24 h after the intervention (*p* value = 0.003), and 6606.5 ± 298 and 19,278.3 ± 743 plts/μL 48 h after the intervention (*p* value = 0.002) (Figure 4).

### 3.5. Transfusion and Adverse Events

Blood transfusions were required in 2.85% (*n* = 2) of patients in the TXA group and 10% (*n* = 7) in the control group. Given the low number of transfusion events (two in the TXA group and seven in the control group), Fisher's exact test was applied and revealed a statistically significant difference between groups (*p*=0.037). Foley catheter replacement was required in 5.7% of patients in both groups.

One patient (1.42%) in the TXA group developed PE, which was clinically diagnosed and successfully treated. No thromboembolic events were reported in the control group. Although the overall incidence was low, this event underscores the importance of thromboembolic monitoring in patients receiving antifibrinolytic agents. No mortality occurred in either group during the study.

### 3.6. Linear Regression Analysis

Linear regression analysis for predicting 24- and 48-h Hb levels and drop revealed that variables such as volume of irrigation fluid, age, and group (TXA and control) accounted for 70.5% and 86.5% of the variance in drop in 24- and 48-h Hb levels, respectively. Specifically, the volume of irrigation fluid had a 97% and 81.4% negative effect, the group had a 76% and 66.7% negative effect, and age had a 91% and 54.5% positive effect on 24- and 48-h Hb drop, respectively. This indicates that higher irrigation fluid volume correlates with lower Hb drop, while increasing age correlates with a more significant drop in Hb levels. Moreover, drug usage in the groups contributed to a reduction in the drop in 24- and 48-h Hb levels (Table 3).

## 4. Discussion

Although TXA has been previously studied in various prostate surgeries, such as transurethral resection of the prostate (TURP) and robotic-assisted prostatectomy, our study adds valuable evidence by assessing a structured local multidose TXA protocol specifically for patients undergoing open simple prostatectomy due to BPH. This approach emphasizes localized antifibrinolytic exposure, which may offer benefits for blood conservation and enhance patient safety.

TXA is widely recognized for its efficacy in reducing blood loss in a variety of surgical disciplines, including orthopedic, cardiac, and obstetric procedures. Its antifibrinolytic properties are particularly advantageous in high-risk surgical scenarios and in patients with coagulopathies or liver disease [1]. The incorporation of TXA into surgical protocols has been associated with reduced transfusion rates, fewer transfusion-related complications, and improved recovery outcomes [2]. Multiple studies and meta-analyses have also confirmed its favorable safety with no significant increase in thromboembolic events—supporting its continued use across diverse surgical specialties [2]. Our findings align with this growing body of evidence, especially in the context of open prostatectomy.

### 4.1. Intraoperative and Postoperative Blood Loss

Our study demonstrated a significant reduction in intraoperative blood loss in the TXA group, consistent with findings from numerous previous studies. Meta-analyses by Ker et al. [11] and Longo et al. [9] reported a 30%–35% reduction in blood loss across surgical specialties, including urology. In urologic settings, studies by Mohammadi Sichani et al. [12], Meng et al. [13], and Kumsar et al. [4] showed decreased bleeding during both TURP and open prostatectomy. TXA has also shown efficacy in reducing blood loss in surgeries involving patients with hemophilia, as well as in orthopedic, gynecologic, and cardiac procedures [14–16].

TXA has similarly been effective in reducing postoperative blood loss in knee arthroplasty [17], spinal fusion surgery [18], and prostatectomy surgery [6, 19, 20]. Cardiothoracic studies also support its use during coronary artery bypass graft [21] and other cardiac surgery [22]. Mechanistically, TXA prevents the conversion of plasminogen to plasmin by blocking its lysine-binding site, thereby stabilizing fibrin clots and reducing blood loss.

### 4.2. Blood Loss Relative to Prostate Weight

A significant difference was observed in blood loss per gram of the resected prostate tissue between the TXA and control groups. This finding suggests TXA's efficacy even when adjusted for gland size. Balik et al. reported no significant differences in the parameter which partially aligns with our findings depending on the timeframe analyzed [23].

### 4.3. Transfusion Requirements

Our results show that TXA significantly reduced the need for blood transfusion. This is consistent with studies by Longo et al. [9], Pourfakhr et al. [20], and Crescenti et al. [24] which reported a decreased incidence of blood transfusion among prostatectomy patients treated with TXA. In cardiac surgery, Abrishami et al. demonstrated that topical TXA use could save one unit of blood per patient [25]. However, Miller et al. found no significant association between TXA and the transfusion reduction [26], a result that partially aligns with some findings in our study. Overall, TXA appears to reduce transfusion needs primarily through reduced intraoperative blood loss.

#### 4.3.1. Operation Time

The administration of TXA in our study did not significantly reduce operation time. While Kumar et al. observed shorter TURP durations due to reduced intraoperative bleeding with TXA, this outcome was not replicated in our open prostatectomy cohort [27]. Differences in procedure type and baseline blood loss may account for the discrepancy.

#### 4.3.2. Irrigating Fluid Absorption

No significant difference in irrigating fluid absorption was found between the two groups. This contrasts with Mohammadi Sichani et al., who reported a reduction in fluid absorption with TXA use [12].

#### 4.3.3. Hb, HCT, and PLT Trends

Although no significant difference in Hb, HCT, or PLT value was observed between groups before surgery or 24 h postoperatively, the drop in this value was significantly less in the TXA group at 24 and 48 h. This suggests that TXA effectively reduces perioperative blood loss. Sichani et al. [12], Pourfakhr et al. [20], and Kim et al. [28] reported similar improvement in Hb, HCT, and PLT trends after TXA administration. Rannikko et al. [6] also noted reduced postoperative Hb decline. In contrast, studies by Mina et al. [19], Longo et al. [9], and Kumar et al. [27] found no significant effect on Hb levels. Balík et al. likewise reported no significant difference in Hb decline at 3 and 24 h postoperatively [23]. These mixed results may be attributed to differences in TXA dosing, timing, and administration route, as well as surgical variability.

#### 4.3.4. Thromboembolic Risk

One patient in the TXA group experienced PE. However, the overall incidence of thromboembolic events was low. Montroy et al., Crescenti et al., and Gupta et al. found no significant increase in thromboembolic complications with TXA use [24, 29, 30]. A meta-analysis by Ker et al. similarly reported no elevated risk of MI, stroke, DVT, or PE [11]. Balik et al. confirmed these findings even up to 3 months postoperatively [23].

While causality cannot be definitively attributed to TXA, this single adverse event underscores the importance of vigilant monitoring, particularly in older or high-risk patients. Although the existing literature largely supports the safety of TXA, further large-scale, long-term studies are needed to fully evaluate its thrombotic risk profile.

#### 4.3.5. Potential Confounding Factors

Several factors can impact Hb levels and the outcomes of recovery. Key influences include initial Hb concentrations, presence of comorbidities like chronic kidney disease or heart failure, and the use of certain medications, such as anticoagulants and iron supplements. Additionally, demographic elements like age and sex, along with nutritional factors—particularly iron intake and donation frequency—are significant. It is essential to consider these variables to ensure that conclusions regarding Hb decrease and recovery postsurgery are valid. Age, in particular, is a crucial aspect when looking at perioperative bleeding and recovery. Though the age gap between groups did not show statistical significance, it was clinically important, so we adjusted for it in our outcome analyses to reduce bias. This adjustment helps enhance the credibility of our results.

### 4.4. Limitation

The relatively small patient sample size is a key limitation of this study. A limited cohort may reduce statistical power and increase the likelihood of both Type I and Type II errors. This could compromise the ability to detect meaningful differences between groups and may obscure important clinically significant findings. Moreover, small sample size limits the generalizability of the results, as they may not adequately represent broader demographic or clinical variations in the BPH population.

This study was also conducted at a single center, which may further restrict the external validity of the findings. Institutional practices, surgical techniques, and patient characteristics can vary significantly across healthcare settings, potentially limiting the applicability of our results to other populations or regions. Another limitation is the short follow-up duration, which restricts assessment of delayed complications, particularly thromboembolic events.

Another limitation is the relatively short follow-up duration, which prevents the evaluation of delayed postoperative complications, particularly thromboembolic events. Additionally, no routine screening for venous thromboembolism (VTE) was performed after hospital discharge, potentially underestimating the incidence of asymptomatic events and limiting our ability to draw firm conclusions about the long-term safety of TXA.

To address these limitations, future research should include larger multicenter trials with extended follow-up periods and routine postoperative screening for thromboembolic events. Such studies would improve statistical reliability and provide a more comprehensive assessment of the efficacy and safety of TXA in open prostatectomy.

## 5. Conclusion

This study demonstrated that the rate of Hb decline at 24 and 48 h postoperatively was significantly lower in patients receiving TXA. These findings suggest that local intraoperative administration of TXA may effectively reduce Hb loss and perioperative bleeding in open prostatectomy.

However, due to the relatively small sample size and single-center design, larger multicenter trials are needed to confirm these results and support definitive clinical recommendations. Additionally, patient age was found to significantly influence postoperative Hb decline and should therefore be considered when planning perioperative blood management strategies, including blood reservation prior to surgery.

## Figures and Tables

**Table 1:** Baseline characteristics, surgical parameters, and perioperative outcomes by treatment group.

Variable	Group		*p* value
	TXA group (*n* = 70)	Control group (*n* = 70)	
Age (years)	60.07 ± 7.44	70.50 ± 6.68	0.234
Weight (kg)	74.8 ± 2.2	80.5 ± 2.7	0.326
Height(cm)	173.2 ± 3.9	176.3 ± 4.5	0.459
BMI (kg/m^2^)	25.04 ± 3.44	26.04 ± 4.22	0.125
Duration of hospitalization (days)	2.50	2.20	0.058
Prostate volume (cc)	97.72 ± 40.86	96.24 ± 34.32	0.817
Weight of resected tissue (g)	16.73	16.54	0.452
PSA (ng/mL)	6.23 ± 4.58	5.83 ± 2.15	0.064
Irrigation fluid (L)	49.57 ± 7.88	50.48 ± 9.24	0.53
Surgery duration (min)	62.11 ± 5.42	57.03 ± 8.12	0.344
Blood loss (mL)	116.65 ± 43.23	210.27 ± 87.94	0.001
Blood loss per gram resected tissue (mL/g)	6.97 ± 0.22	12.69 ± 0.36	0.01
Blood transfusion required (%)	2.85% (2)	10% (7)	0.037
Foley catheter replacement (%)	2.85% (2)	2.85% (2)	—
Anesthesia			
General	0.7% (*n* = 1)	0.7% (*n* = 1)	0.831
Epidural	0% (*n* = 0)	0.7% (*n* = 1)	
Spinal	49.3% (*n* = 69)	48.6% (*n* = 68)	

**Table 2:** Postoperative hematologic profiles at baseline, 24, and 48 h by treatment group.

Variable	Time point	TXA group	Control group	*p* value
Hemoglobin (g/dL)	Baseline	14.12 ± 1.34	14.47 ± 1.56	0.316
	24 h	12.91 ± 1.05	12.28 ± 1.23	0.214
	48 h	11.15 ± 1.54	9.74 ± 1.02	0.02

Hematocrit (%)	Baseline	39.13 ± 3.53	40.52 ± 2.89	0.256
	24 h	37.67 ± 5.65	36.19 ± 4.87	0.162
	48 h	36.42 ± 4.02	31.40 ± 5.75	0.01

Platelet (× 10^3^/μL)	Baseline	190,275.5 ± 59,127	190,519.6 ± 57,319	0.204
	24 h	183,195.1 ± 48,760	179,965.6 ± 50,087	0.197
	48 h	176,588.6 ± 49,654	160,687.3 ± 59,761	0.01

**Table 3:** Comparison of hemoglobin, hematocrit, and platelet drops at 24 and 48 h between the TXA and control groups.

Variable	TXA group (mean ± SD)	Control group (mean ± SD)	*p* value
*Hemoglobin drop (g/dL)*			
24 h	−1.19 ± 0.99	−2.194 ± 1.43	0.0001
48 h	−1.78 ± 0.97	−2.79 ± 1.04	0.0001

*Hematocrit drop (%)*			
24 h	−0.1.46 ± 0.86	−0.4.33 ± 1.53	0.0003
48 h	−1.25 ± 0.92	−0.4.79 ± 1.42	0.0004

Platelet count drop (× 10^3^/μL)			
24 h	−7080.4 ± 326	−10,554.0 ± 489	0.003
48 h	−6606.5 ± 298	−19,278.3 ± 743	0.002

## Data Availability

The data that support the findings of this study are available from the corresponding author upon reasonable request.
